# Automated Bone Age Assessment: Motivation, Taxonomies, and Challenges

**DOI:** 10.1155/2013/391626

**Published:** 2013-12-16

**Authors:** Marjan Mansourvar, Maizatul Akmar Ismail, Tutut Herawan, Ram Gopal Raj, Sameem Abdul Kareem, Fariza Hanum Nasaruddin

**Affiliations:** ^1^Department of Information System, Faculty of Computer Science and Information Technology, University of Malaya, 50603 Pantai Valley, Kuala Lumpur, Malaysia; ^2^Department of Artificial Intelligence, Faculty of Computer Science and Information Technology, University of Malaya, 50603 Pantai Valley, Kuala Lumpur, Malaysia

## Abstract

Bone age assessment (BAA) of unknown people is one of the most important topics in clinical procedure for evaluation of biological maturity of children. BAA is performed usually by comparing an X-ray of left hand wrist with an atlas of known sample bones. Recently, BAA has gained remarkable ground from academia and medicine. Manual methods of BAA are time-consuming and prone to observer variability. This is a motivation for developing automated methods of BAA. However, there is considerable research on the automated assessment, much of which are still in the experimental stage. This survey provides taxonomy of automated BAA approaches and discusses the challenges. Finally, we present suggestions for future research.

## 1. Introduction 

Bone Age Assessment (BAA) often expressed as skeletal age assessment is a clinical method for evaluating the stage of skeletal maturation of a child [1]. BAA is not introducing a new field of skill in medicine science, as the eruption of the second molar was used in the Roman Empire as an indicator for calling young males for military service [2]. In the nineteenth century, age was estimated by dentists and tooth eruption was considered as a reliable method to detect the age of a child. In that era, the minimum bone age was calculated to be 7 years old in Britain [3]. However, some experts have opposed this method for the estimation of age. In 1846, Dr. Pedro Mata announced his concern about estimating age based on only tooth eruption [4]. Rontgen discovered X-rays in 1895 and his discovery made a revolution in the estimation of age for living subjects. This innovation based on radiography of the skeleton was used as a complement to tooth eruption [5]. In 1886, Angerer was the first person who stated that the carpus bone in the hand is an indicator for the estimation of age in young people [6]. The first systematic review of age variations in the carpus bone was published by Behrendsen in 1887 [7]. The researchers tried to define the age of the subject based on the radiologically defined maturation of the hand wrist bone [8]. Between 1950 and 1980, the most important methods for the estimation of age based on radiological analysis of the carpus bone were defined as Greulich and Pyle (GP) [9] and TW [10]. Both manual methods are time-consuming and prone to inter- and intraobserver variability. These are motivation for presenting computerised system of BAA [11, 12].

### 1.1. Bone Age Assessment from Radiographs

BAA is a radiological examination to determine the difference between the skeletal bone age and the chronological age (the real age from birth date) [13, 14]. This discrepancy presents abnormalities in the skeletal growth of children or hormonal problems [15]. To estimate bone age (BA) based on an accurate and reproducible method is not only a difficult process but also a time-consuming radiological procedure [16]. BAA is based on three sequences as follow;

- appearance of primary and secondary centers of ossification,
- growth of both centers,
- timing of fusion of the primary and secondary centers.

The appearance and change in the above processes have been clearly identified in dry bone and radiographic images [17]. The judgment about the age with no doubt based on the identification of timing of the appearance of ossification centers and epiphyseal fusion identification, is dependent on whether the dry bone is being observed or whether it is being visualized by an imaging method, such as a radiograph [18]. The assessment of chronological age is based on a matching process, which includes a comparison of a radiograph image of a subject to a defined reference that involves a sample of known sex and age [19]. The process of age estimation is basically a measure of the biological maturity that is converted to the chronological age by comparison with a reference [20].

Reference data for the age estimation have been collected from various resources and have been presented as a series called an “Atlas” [21]. Much of the data used to compile the atlas were collected during longitudinal studies that took place in the 1900s [22]. These data was collected for each child as part of an anthropometric exercise in the format of standardized radiographs [23]. Since the goal was to show the growth of normal life, all the participants had a health history without any disorder or disease. The data collected provided reference data to estimate the age of an unknown child for identification or medical and educational purposes [24].

Factors, such as the environment, and, especially the nutritional situation, strongly affect the development of children in society [25]. The atlases, which were developed based on healthy children who had adequate nutritional intake, were deemed to be suitable to use as the standard for comparison goals [26]. These atlases included a data set of images, which showed the maturational changes as a powerful source for age estimation in the living [27]. The atlases took the maturation step of a child of unknown age and found the most appropriate age rather than an evaluation of the maturational steps of a child with known age [28]. This is a routine that is commonly used for most of the atlases and the age identified by the expert is based on it accordingly. Using the atlas means that they present a temporary snapshot of the maturation tempo of children of known race [29]. The problem is whether this information is relevant to modern society or whether it can be used for children of different races with a different diet and medical care [30].

The majority of the literature works show that the atlas for estimation of age is based on the left hand wrist [31, 32]. Such research could be classified based on the methods used to test accuracy. These classifications involve the following activities.

- Testing age assessment methods on a special society.
- Comparing of error observer.
- Comparing the accuracy of different atlases from the same skeletal area on the same group.
- Comparing of the maturity levels on different body parts, on the same group.

Although, assessment of bone age is possible from many bones in the body such as elbow, pelvic, clavicle, foot, shoulder, or ankle, however, the high costs, long interoperation, and the risk of exposure to radiographs show that this is neither suitable nor practical for researchers to use for BAA [33].

### 1.2. The Need for Age Assessment

In 2010, UNICEF stated that just around 50% of children below five years in the developing countries have birth registration documents. For example, 64% of births in sub-Saharan Africa and 65% of births in South Asia are unregistered, [34]. This issue deprives children from their original rights. Without any evidence to indicate their age, children are at risk of underage recruitment into fighting forces and early marriage [35]. They are more vulnerable to judgment as an adult rather than a child or juvenile in criminal courts or in looking for international protection as an asylum seeker [36].

Children without any ID or birth document not only have less chance for leniency in sentencing and the benefit of the facilities in juvenile rehabilitation centres but are also treated as adults for issuing penalties in law enforcement [37]. When a juvenile is wrongly identified as an adult, it could change his/her life in consideration of the maturity, capacity, or ability in reintegrate. When a child is incorrectly classified as an adult, the child is put at risk of a cycle that is disproportionate to the child's situation, age, or maturity [38]. Children deserve special protection and are below the age of criminal responsibility and may enter the formal justice process through incorrect identification [39]. Hence, a realistic definition of age is crucial to decide and treat children and juveniles properly and unregistered migrant children are at risk of abuse and discrimination [40].

Unregistered or migrant children are vulnerable to many kinds of prejudice and injury. In 2007, some refugee unregistered children in Guinea were arrested arbitrarily by police under the law enforcement as adults, and they were unable to assert their age. Refugee children in Europe had a similar position [41]. They have been entered into the adult asylum determination process because their age was not clear. They were deprived from any concessions that are of benefit to children. In the UK, this position means that they have more limited rights for the asylum interview, do not benefit from a lawyer to support them at the interview, and are even detained during the decision process [42]. Being considered as an adult provides the refugees with special facilities and financial assistance. Positive decisions have occurred through national campaigns to register the birth date of children [43]. Afghanistan and Bangladesh have created their first government birth registration systems, while India and Pakistan have tried to promote birth registration in Asia [44].

At present, there is no agreement in European countries on a specific method for age estimation of a supposed minor. However, some countries such as the United Kingdom use an interview to determine whether or not individuals are minors. A “Merton compliant age assessment” performed by two trained expert social workers is the accepted method for the UK Border Agency. Austrian authorities use a “multifactorial examination methodology” type assessment to determine individuals' ages. This assessment consists of three factors or evaluations: an examination by a doctor, X-ray testing, and a dental analysis. France applies a psychological interview for age assessment of unaccompanied minors [45].

Recently, ACNUR (Alto Comisionado de las Naciones Unidas para los Refugiados) recommended that European Union (EU) authorities unify their techniques for age assessment to defend the human rights of immigrant children. Some international communities of specialists such as the American Board of Forensic Odontologists (ABFO) and the German Study Group of Age Estimation and Legal Medicine (AGFAD) have released their instructions and guidelines for bone age assessment [46].

Despite this development and the attempts by UNICEF and other international organizations, many children still do not have registration documents. Therefore, when a government or any agency wants to estimate the age of an unregistered child they need a secure and accurate method to assess the age [47]. Hence, an automated BAA system plays role as an important tool for a secure method in the clinical environment with an easy way to use in BAA [48].

The remainder of this paper is organized as follows.  Section 2 presents taxonomy of existing system in BAA. We discuss the limitation factors and challenges in BAA methods in Section 3. Finally, future research direction is presented in Section 3.1 and paper the is concluded in Section 4.

## 2. Taxonomy of Methods for BAA

### 2.1. Automated Approaches in BAA

As aforementioned, bone age is defined as an indicator of skeletal maturity using radiography of the ossification center. Despite a large volume of scientific research on BAA, there is a lack of agreement concerning the accuracy of bone age methods which is acceptable for a clinical environment [49]. For BAA in both clinical environments and courts of justice, it is important to yield the most accurate result. An automated bone age system could reasonably eliminate the role of a human observer, which would decrease the subjectivity in assessment as the main reason for the loss of accuracy [50]. This part of the survey classifies the computerized methods for BAA, which is the significant topic of this research.

Most of the automated systems for estimation of bone age derived the state of skeletal maturity from X-ray images of the left hand wrist [51]. This is not an easy task because the hand wrist includes a group of various bones, which rapidly change shape over time, and also some bones overlap with maturation [52]. As mentioned previously, analysing bone age is a complicated process even for experts [53]. Most computer-based methods use the TW approach due to the scoring for skeletal maturity and separate stages. Specific image processing techniques are needed to assess the radiograph of a known hand [54].

Researchers admit the significance in automating the method for the estimation of bone age. These methods use some intelligent techniques, such as segmentation of the hand, while some are only used in the research environment [55, 56]. It is estimated that computerized methods in BAA could decrease the cost of assessment of bone age through a decrease in the time that radiologists spend in predicting the bone age [57].

#### 2.1.1. HANDX System

The first semiautomated system for BAA was introduced by Micheal and Nelson in 1989. The authors claim that this system, which they call HANDX, is able to automatically segment bones in X-ray images of the hand wrist using image processing techniques [58].

This system reduces the variability of the observer and the output is useful to detect abnormalities of skeletal growth in children. This computer vision system works in three parts: preprocessing, segmentation, and measurement. In the first stage the radiographs are normalized to feed in the second step. The segmentation stage finds the specific bones in the hand and also isolates the edges of the bone, and, finally, quantitative parameters are achieved in the last stage. This semiautomated system has no reasonable accuracy when the hand image is fused and has never been evaluated on a large scale.

#### 2.1.2. PROI-Based System

In 1991, Pietka and his research group developed a method based on PROI analysis. PROI is the region that includes the phalanges and epiphyses [59]. For the estimation of bone age, in the first process the system scans a horizontal line and the lower boundary of the PROI is found before the soft tissue between the thumb and first finger is detected. In the next stage, the upper boundary containing a horizontal line at the edge of the third finger is scanned. When the upper, lower, left, and right boundary of the PROID have been detected, the segmentation stage starts. A gradient image is used for segmentation of the bones and the output threshold is based on empirical analysis to determine the bone edges. The density of value of pixels at the end of the region is higher than the center section. In this method, the boundary between the third distal, middle, and proximal phalanges is measured. This measurement uses the standard table prepared by the Garn group [60] involving phalangeal length to convert into skeletal age. The system has been evaluated by 50 computer radiographs (CR) of patients and a comparison of the results with an observer (radiologist). The mean difference yielded from the evaluation was 0.02 mm with a measurement error of 0.08 mm [61].

#### 2.1.3. The CASAS System

In 1994, Tanner and Gibbons proposed a computer-based skeletal age scoring system (CASAS), based on the Tanner and Whitehouse2 (TW2) method using radius, ulna, and short bones (RUS) [62]. This semi-automated system digitized X-ray images with a light box and monochrome video camera. Every bone is located on the digital camera using an overlay pattern. The computer assesses the bone age by matching and finding the best average based on fast Fourier transform. The result minimizes the root-mean-square error between the coefficients of the Fourier transform from the unknown bone and Fourier transform of the available bone templates. The patterns are produced by averaging the Fourier transform coefficients using 10 images from bone stages. The system improves to five-root-mean square by using a Gaussian function. The images are only applied to develop a standard skeletal maturity for TW and not for developing the actual bone scoring style [63]. However, in the system the templates have a vital role and selection of the source for making the template is very important. The CASAS system has been tested and evaluated using X-ray images from children in normal and a stable pathologic position. There has been some research on a comparison between the CASAS method and the manual TW method and the results present a reasonable assertion that the CASAS estimation is more accurate than the manual TW method. Frisch et al. [64] stated that the common conclusion about the CASAS system is that it presents a suitable method for BAA for children in a normal situation. The system is based on a very simple image processing process and the method allows repeatability. The most important weakness is that the method does not work for assessing pathological problems due to deformation of the bone. The method also decreases the assessment objectivity because of the huge number of manual interventions.

#### 2.1.4. Middle Phalanx of the Third Finger Based on an Active Shape Model

Niemeijer developed an automated system based on the TW2 method that classified the middle phalanx of the third finger utilizing the active shape model [65]. The model uses the mean object, description of modes of variation, and a covariance matrix for statistical measure. In this method, the radiologist specifies the third phalanx and the computer segments the bone with the active shape model. The matching function is executed by the highest relationship between the pixel scale for RIO (region of interest) of the unknown bone and the pixel scale from the sample images. The accuracy of the system investigated was 73% to 80% compared to an observer. The drawbacks of the system are two parts: first, this system only works for stages of E to I in the TW method, and, secondly, the system only covers the age assessment for ages between 9 and 17 years.

#### 2.1.5. Neural Network System Based on Linear Distance Measures

In 1995, another system was developed by Gross, using a user to measure the features from the hand wrist radiograph and a decision system based on neural network to assess the bone age [66]. The system started with ten measurements but using linear regression analysis it gave better correlation coefficients and selected seven measurements. The weakness of this method is that the system does not use the morphological features applied in the GP or TW methods. Hence, there is no major difference between using the neural network method and using the manual GP method for BAA.

#### 2.1.6. Phalanges Length Based System

The first version of a fully automated system was released in the 1990s based on a Picture Archiving and Communication System (PACS), which uses the digital atlas of radiographs in a controlled manner [67]. This system applies a rough estimation based on the phalangeal length measurements taken from phalangeal length tables prepared by Garn. The system extracts specific regions of the hand wrist based on the rough estimates. For example, if the subject's age is less than eight years, the carpal bone region is selected for analysis. The image processing techniques and algorithms of the system used to evaluate and retrieve the skeletal features are very simple but time-consuming. The system uses a web-based image distribution with a digital atlas using a query language engine. However, this method is introduced as a practical and reasonable computerised means of BAA using applied fuzzy classification to cover the noisy data and subjective decision. The fuzzy systems are dependent on the reference population group because of using the relationship with age rather than measuring the skeletal maturity, which is a significant restriction. Therefore, most of the test results released for the system are based on the accuracy of the region of extraction or segmentation; a comparison between the estimated age by the system and the chronological age shows roughly a year's difference. The method tries to improve the segmentation of the phalangeal epiphyses by applying Gibbs random with contour model segmentation, to improve the carpal bone analysis and radial epiphysis. BAA using the phalangeal length always raises the question of what happens to the estimation if the preliminary assessment is inaccurate, which is the main drawback of this method, and, hence, the phalangeal length is not a reliable indicator for skeletal maturity.

#### 2.1.7. The Third Digit: Three Epiphyses—Sato et al.

Sato et al. proposed an automatic system to assess the bone age for Japanese children based on analysing the bones of the third digit [68]. This system is known as the computer-aided skeletal maturity assessment system (CASMAS).

This method uses the proximal, middle, and distal epiphyses of the third digit based on the widths of the epiphysis to metaphysis and width of the metaphysis-epiphysis to the metaphysis and evaluates the radial epiphysis when the epiphyses is complete. Evolution of the system has presented reasonable results for the age range between 2 and 15 years, but for very young children and those above 15 years, the results are not so accurate. This is because the epiphyses is under development in the young children and overlaps for older children. To a certain extent this problem limits the use of this system for BAA.

#### 2.1.8. Phalanges, Epiphyses, and Carpals

The National Tsing-Hwa University in Taiwan presented a computer-based system for BAA, based on the third digit; however, this system involves the process of extracting the left hand from the X-ray image from both hands on the same radiograph [69]. This method utilizes thresholding methods and heuristic searches to rotate the radiograph in the preprocessing stage. The system works on the phalangeal region of interest (PROI) and segments the phalangeal bone with Gabor filters for smoothing and Canny edge detector as well as local variance method for finding the edge and refinement. The PROI segmentation includes full grey-scale information that shows a successful method for BAA with low error rate in evaluation.

Two series of information are derived from the PROI segmentation. The first set containsgeometric indicators of length, width, and area of distal and proximal phalanx. Despite the length of the distal phalanx having low contrast in some X-ray images, it has been utilized to normalize the lengths and the areas. The second set includes the information of the epiphysis shape of the distal phalanx. These features are fed to the neural network for analysis. The method uses three neural networks for leave-one-out statistical training and testing, including

- back propagation,
- radial basis function,
- support vector machine.

Although the support vector machine has the best performance among these three networks, its accuracy was evaluated to be 85%. To decrease the error rate, the system applied the carpal bone information for subjects below 8 years using a fuzzy membership function. The carpal bone age is considered as a mask for output value of the neural network and the final result combines the carpal age result, plus 2 outputs above as well as 2 outputs below the estimate. If the children are older than seven years, the two largest neural network estimates are applied. The research group improved their method, in 2008, by screening Turner's syndrome using measured bone age and distal-middle phalanx ratio.

#### 2.1.9. Mahmoodi Model

The above mentioned researchers used computed features. Mahmoodi [70] proposed an automated system based on analysis of the phalangeal utilizing an active shape model and knowledge based technique. The system applies a hierarchical search to focalize the bones and then an active shape model is performed by a bone contour. The system extracted three shape features that had 0.72 and 0.89 correlation coefficients with actual age. These shape features include moment of the proximal end of the phalanx with the ratio of width of epiphysis to the metaphysis. This method identified a reasonable relationship between the epiphysis-metaphysis region and chronological age. They reduced the risk in assessing the bone age by using the Bayes risk principle from the decision theory. The system has been evaluated with a leave-one-out technique. In this technique one X-ray image is removed and the system is continued using the remaining images and trained with the current sample, then the removed image is evaluated as a new parameter. The researcher presented the accuracy of the system as being 82% for male patients and 84% for female patients. They claim that they could increase the accuracy of the system by improving the training set.

#### 2.1.10. Neural Network Classifiers Using Features of the RUS and Carpal Bones

Liu et al. [71] developed a computerized system for BAA using an artificial neural network based on two geometric features of the RUS (radius, ulna, and short bones) and carpal bones. This system uses a huge database of samples and algorithm of particle swarm applied for segmentation of the bones. This method applies two classifiers to estimate the bone age: the first one is RUS bone and the second one is carpal bone for samples below nine years old. This method has a small standard deviation of the differences when comparing the system and observer. The positive point of this system is that it decreases the variability in the carpal bone-based system compared with the previous systems.

#### 2.1.11. Neural Network Based on the Radius and Ulna

Vega and Arribas [72] proposed a computer-based system to predict the bone age based on the TW method and using the radius and ulna. This system is assisted with manual landmarks and then applies an adaptive clustering technique for segmentation of the radius and ulna. The method applies neural networks in the decision state to make a posteriori probabilities that predict the error rate; this feature is specific to this method. The range of the mean difference of the system and observers is large and this method is limited to just four TW3 levels. However, the researchers claim that their method could be extended by improving the bone segmentation. This method proposes that a neural network is valuable for further investigation.

#### 2.1.12. Neural Network Analysis Based on the Epiphyses and Carpal Bones

The common process for assessment of bone age of the hand wrist bone is to make an outline of the border of the bone and then extract the feature from the outline. For the carpal bone, it is too hard to discover the bone border due to the low contrast in the edges, noise in X-ray image or overlapping soft tissue. Rucci with his associates [73] stated that they could overcome this issue by using a trained neural network that extracts features of images. This method uses an attention focuser and a bone classifier in a neural network architecture. The attention focuser implements pixel processing that links a hidden neural network to create an output, which they call X and Y value relating the centroid of the bones of images. The method was tested with 56 radiographs of low quality and 16 extra images. The results demonstrate 65% accuracy and 97% accuracy, respectively, with 0.85 years for standard deviation. The results present the neural network as a useful technique for classification in the TW2 method. The system was improved to present a fully automated method for age estimation. In this method, a user labeled specific regions of the bone on the radiograph. The pixel-processing technique is the same as the Rucci method except that it contains the epiphyses analysis. This manual labeling method claimed 0.05 years for average difference and 0.7 for years standard deviation between the estimated age and observer and 1.4 years for error rate. The published results are reasonable but the age ranges used for testing the system are not presented that the system is applicable for missing data or overlap bones. The method introduces the neural network as a powerful technique for image processing. However, the main drawback is that the neural network system starts in a dumb state.

#### 2.1.13. The Royal Orthopaedic Hospital Skeletal Ageing System

Hill and Pynsent [74] described Royal Orthopaedic Hospital Skeletal Ageing System (ROHSAS) based on the 13-bone and 20-bone TW2 method. The system works with an iterative method and finds the hand outline, the phalanges, carpus, and radius-ulna bone and estimates the bone age in about four minutes. This method is also able to detect the left and right hand using radius and ulna widths. A fuzzy set and entropy technique is executed for bone segmentation. A shape recognition method is used for bone classification with normal fuzzy and fractional fuzzy grammars and an octal chain code defined by Kwabwe [75] that specifies the bone edges. The user has the facilities to interpose or ignore the bone classification if needed, such as the CASAS system. Cox [76] tested the system with 98 images from the International Children Centre London Longitudinal Study. The results released show 0.5 year between the system and in 74% of the estimation there was no difference between the system and observer; however, the system has a 25% rejection rate. As a result, Cox stated that the system is a reliable method for BAA albeit there is a need for a larger group of sample images including the normal range for evaluation.

#### 2.1.14. BoneXpert System

The BoneXpert system is another automated method for BAA that was proposed in 2009. This method works based on shape-driven active appearance and the TW RUS-based approach (using the radius, ulna, and short bones) [77]. The shape and intensity features make a robust algorithm of the active appearance model. A set of components of more than 3,000 bone contours are rotated and scaled, based on the Gobar filters which the parameters are formed in the active appearance model. Thirty coefficients were chosen for features of images using a linear regression technique fed into the active appearance model. Although the usability of the system is still under evaluation, preliminary testing shows that the performance is reasonable and that the accuracy is stated as 0.42 years for using the Greulich & Pyle (GP) method and 0.80 years for using the TW2 method. The rejection rate of the system was about 1% for poor quality but it increased to 18% in some cases for the radius and ulna. The specific point of this method is that it assesses the accuracy of the bone age utilizing the relationship between the X-ray image and linear growth. The standard deviation calculated was 0.5 years, which showed a jump in reproducibility calculated by automated method. The BoneXpert system has been published as a commercial package since January 2009.

#### 2.1.15. Automated Web Based System Using Histogram

In 2012, Mansourvar et al. [78] developed a fully automated BAA system that uses compression techniques based on the histogram techniques. This approach works on an image repository and similarity measures and uses a content-based image retrieval (CBIR) method for image processing. The system includes a knowledge base consisting of 1100 hand X-ray radiographs classified by gender as well as ethnicity. This approach overcomes the segmentation problem by utilizing a histogram that is further elaborated upon in Section 3. The evaluation presented 0.170625 years for error rate of the system thereby indicating that this method is a credible method for BAA. However, the system is not reliable for images with poor image quality or abnormal bone structure.

All the mentioned methods discussed under the category of automated approaches in BAA are presented in Table 1 chronologically. The methods have been compared with their specific features debated in Section 2.1.

### 2.2. Summary

The review of automated approaches in BAA presented in Section 2.1 stated that it is possible to obtain an accepted error rate without human intervention. Some of the automated approaches focus only on a small set of bones in their processes, for example, the third digit method. Most of the automated methods utilized the radius, ulna, and short-bones (RUS bones).

However, the range of selection of the bone zone in the hand wrist area could be extended even in normal subjects to reduce the risk of variability of unreliable bone age estimation within bone maturity across the hand [79].

Table 2 presents a comparison of accuracy and error rates between some automated approaches in BAA that have acceptable error rates. The accuracy results of these systems justify the significance of the methods used.

## 3. Discussion and Identified Problems

**Figure 1:**
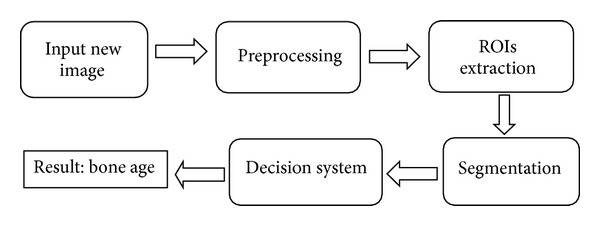
A general model of the automated bone age assessment systems.

With the huge volume of demand for BAA, a major shift towards automated methods is inevitable. Automating the assessment of age in clinics speeds up the process of human identification and saves money [80]. The computerized systems for assessment of bone age have been explained in 15 classifications. In the first 14 systems, the common process is image preprocessing, extraction of RIOs, image segmentation, making decision, and getting a result.  Figure 1 shows a general model for the systems. This is only one simple model based on the review in Section 2.1.

An automated system for the assessment of bone age started with a digital radiograph of the left hand, or in the format of digitization of X-ray images [81]. The image of the hand in the form of an X-ray is inserted into the system by the user or radiologist. The preprocessing stage depends on the subsequent stages in the system starting after uploading the radiograph [82]. Usually, the preprocessing step prepares the images for further analysis, for example, removing the background of images or rejecting the images with low quality for processing [83]. Most of the algorithms were based on a small set of bones in features analysis and caused more risk for BAA. Although the region of bones can also be extended, even for normal children, it increases the terrific load in image processing; in addition, the accuracy in respect of legal status is a consideration due to the limitations in segmentation techniques [84]. The review concerning the effort of current research on computerized BAA has shown that all the methods (except the last one) focused on the technique of extraction of ROIs (region of interest). The basic goal of extraction of ROI level is the parse assessment of bone age into separate stages. Different methods use different regions, such as the carpal bones, phalangeal, or the radius and ulna epiphyses. The output of this stage is a special region including the interest area in the hand image [85]. The ROI extraction stage presents the main challenge for the current automated system: Image segmentation.

Image segmentation is a complicated process because there is no standard routine or definition for it, nor is there a unique standard method for its implementation [86]. The segmentation process is defined to separate the specific region of other regions of the hand and is performed based on the different attributes in the X-ray, such as intensity or bone texture. The segmentation process is implemented by a contour of bone edge or bone region. The performance of this counter is a variable based on the extracted features. This is the reason why the number of algorithms based on the hand wrist presented in the literature suffered the problem of segmentation of special regions in the X-ray image, and the lack of sufficient image processing techniques leads to low accuracy of assessment of bone age [87].

**Figure 2:**
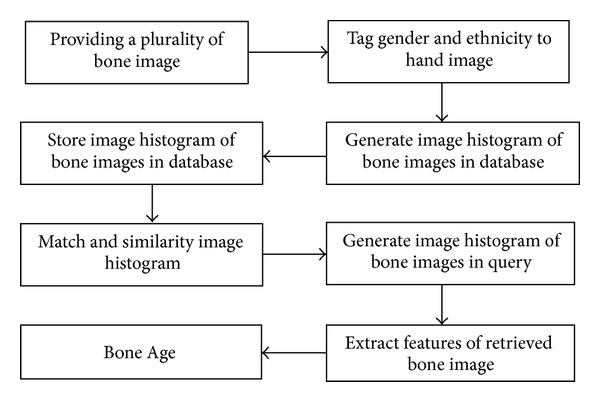
System procedure for bone age assessment using histogram technique.

The last method in the automated approaches [78] generated histograms, which involves resizing of the images. This method provides a new image processing technique to assess the bone age. It is believed that each X-ray image of the bone is unique and also has a unique corresponding image histogram. The histogram of the image is used as an indicator to detect the unknown bone image. The image is tagged with a corresponding profile and stored in a database. This database would be used as a reference to compare bone images of unknown profile with bone images of known profiles. To assess the bone age, a corresponding image histogram of the radiograph is created and compared with the histograms stored in the feature knowledgebase. The age of the bone is estimated by the closest matching with the image histogram of other bone images. This method overcomes the segmentation problem of prior research.  Figure 2 depicts the procedures of the proposed work.

Another major challenge still observed in the current automated BAA systems is that they rely on the left hand wrist bones from normal cases with suitable quality [62, 66, 67, 77, 78]. Hence, there is no solution for cases that have a defect in their hand because of unexpected incident or injury and also for people with bone abnormalities in their hands. This survey shows that there is no automated solution for noisy images or missing data of hand radiographs for any reason. The main problem of all the above methods is that they are not reliable methods for an assured BAA, because they do not facilitate estimation of bone age for all needed cases.

### 3.1. Future Direction

Although it is obvious from the papers reviewed in this survey that there is significant efforts for automated assessment of bone age, a number of challenges still exist in the available computerized system and there are big gaps for improving the BAA systems. The main gap, as mentioned in Section 3, is the lack of research on the role of other bones in the BAA system and the contribution these bones can make in respect of missing data for automated assessment.

It remains ambiguous whether using other bones in the body in the computerized method can be helpful for radiologists when the image of the hand is not available for any reason. Hence, maybe developing an automated system for BAA based on hybrid atlases can be useful to cover this problem and to achieve a better output.

The Group on Forensic Age Diagnostics in Germany advised that forensic age estimation should be performed using hybrid evidence for more confident results such as using X-ray of the left hand or teeth examination or physical examination to ensure that the subject has reached the legal age or should be considered as a minor [88]. Although there is no adequate method for age assessment, using a combination of various methods will reduce the error rate. Nevertheless, this branch of science needs more investigation to achieve more accuracy and a precise standard method for bone age assessment based on a hybrid approach.

## 4. Conclusion

Recently, BAA has attracted considerable academic interest. BAA is regularly applied to evaluate growth, management of limb length discrepancies, scoliosis, and the diagnosis of endocrine disorders and generic disorders in children and juveniles. The manual methods used to determine age are often time-consuming and imprecise. Hence, there is an increasing need for automated methods in determining the age of an individual with more precise results.

In this paper, we presented a comprehensive survey on the computerized methods for BAA. It is expected that an automated system would improve the accuracy and precision of BAA in both clinical and research practice. In spite of the fact that the volume of automated methods for BAA has increased, they are still in an early phase of development. Noisy images and incomplete data or poor contrast of some sections in hand images are the critical problem for automated BAA. The implementation of hybrid systems and using various standard skeletal atlases as references could address the problem of assessment due to the limitations in the automated BAA method using hand radiographs.

## Figures and Tables

**Table 1:** A comparison of automated approaches in BAA.

Approaches	Year	Inventor	Method	Advantage	Disadvantage	Reference
HANDX system	1989	Micheal and Nelson	Segmentation and isolated	Reduced observation variability	No reasonable accuracy	[58]
PROI-based system	1991	Pietka et al.	Segmentation of phalanges and epiphyses	Low mean difference and error rate	Evaluated in small scale	[59]
The CASAS system	1994	Tanner and Gibbons	Based on the TW2 RUS method	More accurate than manual TW method	Did not work for assessing with pathological problem	[62]
Middle phalanx of the third finger	2002	Niemeijer	Segmentation of middle phalanx of third finger utilized the active shape model	Accuracy of 73% to 80% compared with an observer	Only covered the children between 9 and 17 years	[65]
Neural network system based on linear	1995	Gross et al.	Based on linear distance measures	Better correlation coefficients	Did not use morphological feature	[66]
Phalanges length based system	1990	Pietka et al.	Segmentation of phalangeal length or carpal	Reduce subjective decision	Depends on the reference population group	[67]
The third digit-three epiphyses	1999	Sato et al.	Analyzing the bones of the third digit	Reasonable accuracy	Covered the children between 2 and 15 years	[68]
Phalanges, epiphyses, and carpals	1999	Hsien et al.	Based on phalangeal region of interest (PROI)	Low error rate	Poor image processing techniques	[69]
Mahmoodi model	2000	Mahmoodi et al.	Analysis phalangeal and active shape model	Reduced the risk in assessing the bone age by using the Bayes risk principle	Capability of further progress	[70]
Neural network classifiers using RUS and carpal	2008	Liu et al.	Based on RUS and carpal bones	Small standard deviation of the differences	High image processing loading	[71]
Neural network based on the radius and ulna	2008	Tristàn-Vega and Arribas	Adaptive clustering technique for segmentation	Improving the bone segmentation	Limited to four TW3 levels	[72]
Neural network analysis based on the epiphyses and carpal	1995	Rucci et al.	Based on the TW method and using the epiphyses and carpal	Useful technique for classification in TW2 method.	Neural network system started in dumb state	[73]
The royal orthopaedic hospital skeletal ageing System	1994	Hill and Pynsent	Based on the 13-bone and 20-bone TW2 method	Reliable method for BAA	Small group of sample images	[74]
BoneXpert system	2009	Thodberg et al.	Based on shape driven and the TW RUS based	High accuracy	Rejects images in poor quality	[77]
Web-based system using histogram	2012	Mansourvar et al.	Based on the histogram technique	Remove the segmentation method	Not reliable for images with poor image quality or abnormal bone structure	[78]

**Table 2:** Comparison of accuracy (error rate) between automated approaches in BAA.

Number	Comparison of error rate in years	
	Method	Error rate
1	Mansourvar et al. [78]	Error rate is about 0.170625 years
2	Thodberg et al. [77]	0.42 years for using the GP method and 0.80 years for using the TW2 method
3	Hill and Pynsent [74]	Error rate is about 0.5 years
4	Rucci et al. [73]	Error rate is about 0.7 years
5	Mahmoodi et al. [70]	Bone age accuracies of (82 ± 3) % for males and (84 ± 3) % for females
6	Hsien et al. [69]	The accuracy was evaluated to be 85%
7	Pietka et al. [67]	Error rate is roughly 1 year
